# Supplementary figures and images for: Development of microfluidic chip for entrapping tobacco BY-2 cells
Source: PLoS One. 2022 Apr 14;17(4):e0266982. doi: 10.1371/journal.pone.0266982 (PMC9009702; doi:10.1371/journal.pone.0266982)

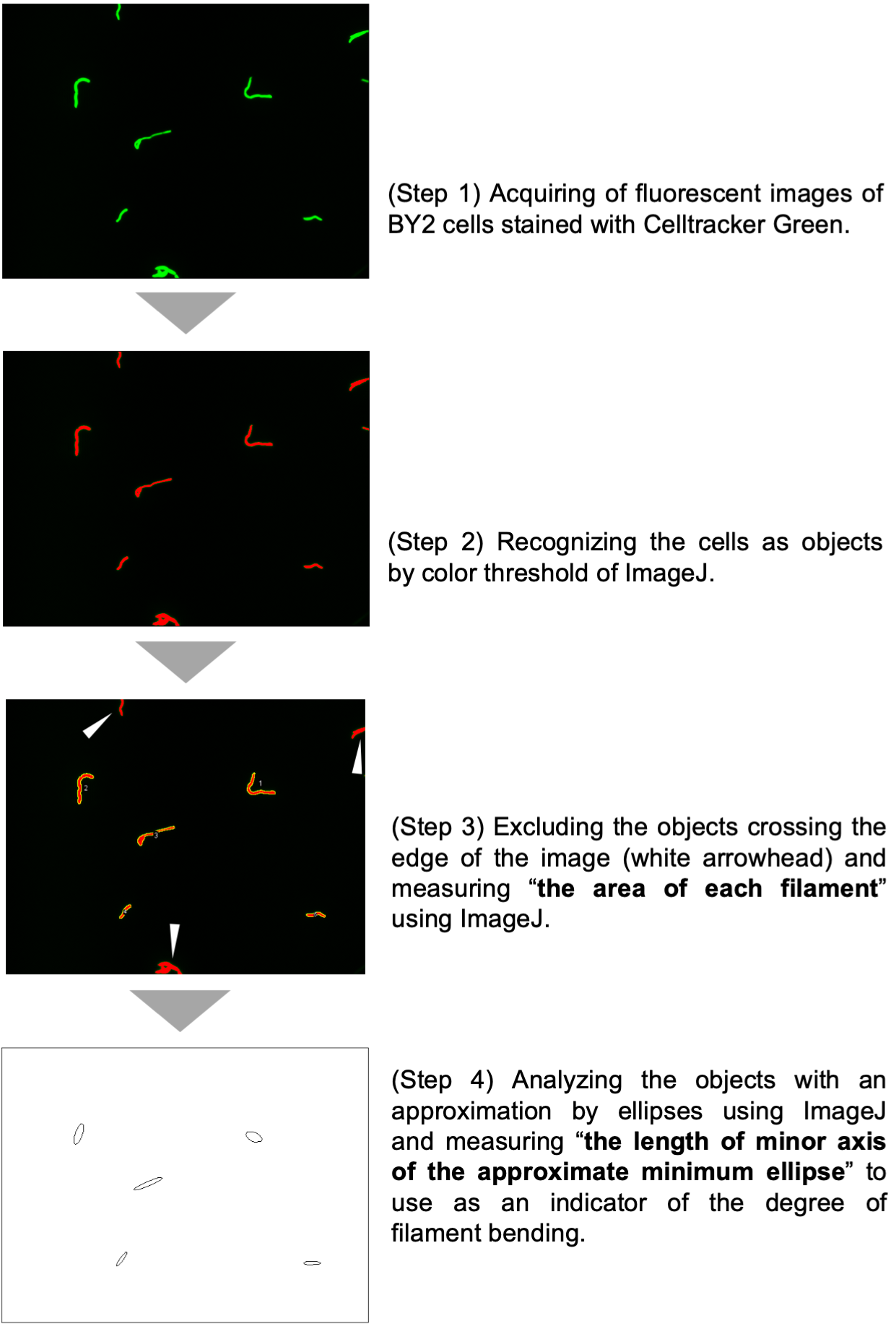

Supplement: S1 Fig — (TIFF) [file pone.0266982.s001.tiff]

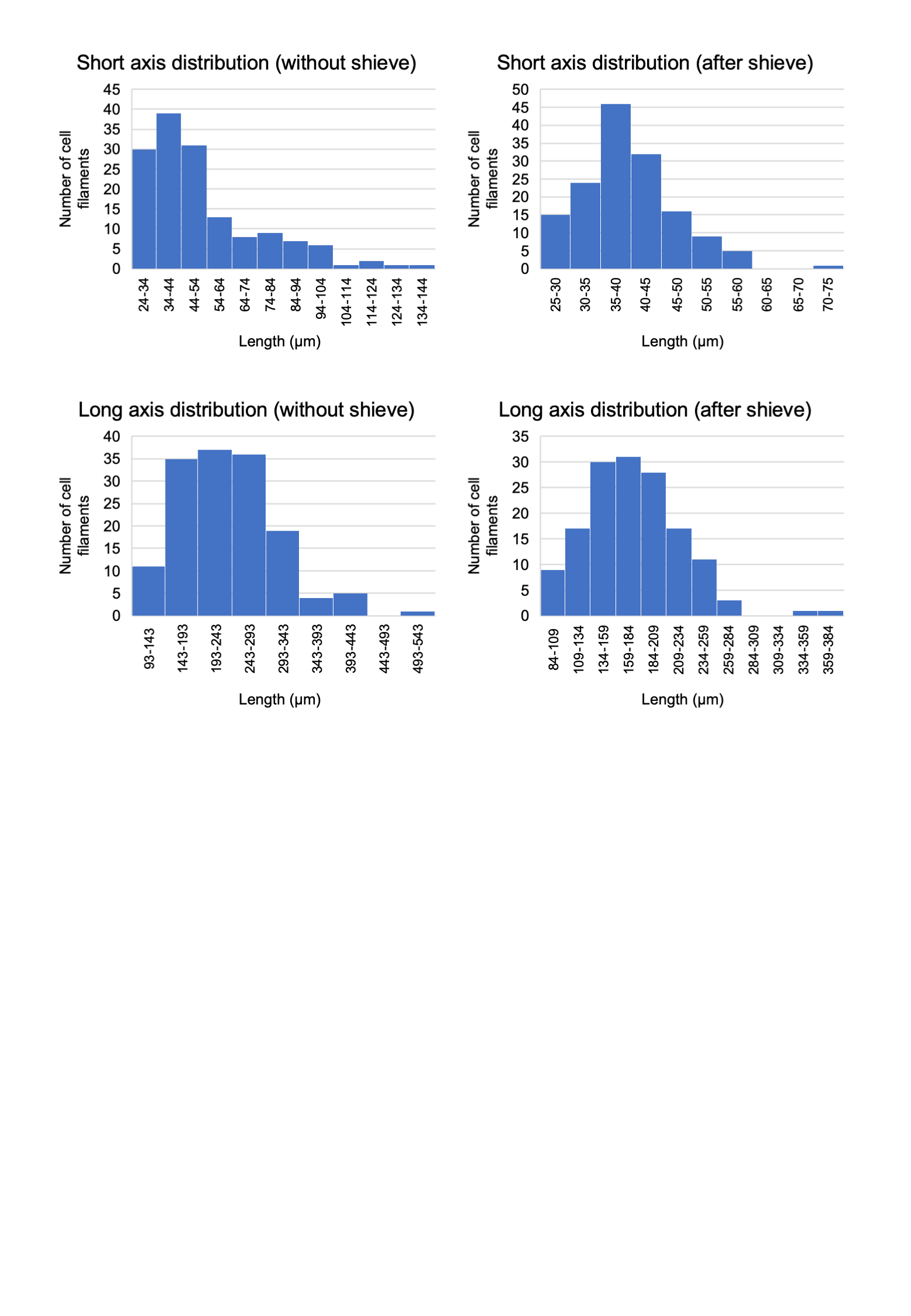

Supplement: S2 Fig — (TIFF) [file pone.0266982.s002.tiff]

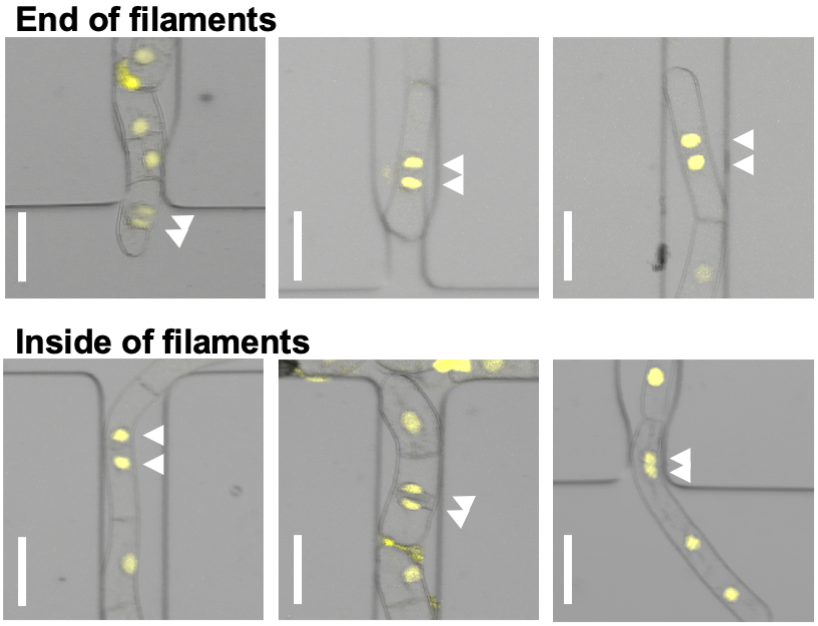

Supplement: S3 Fig — BY-2 cells expressing 3xVenus-NLS were observed. Arrowheads indicate the nuclei in anaphase or telophase. LSM images were merged from 8 consecutive optical sections. Fluorescence images were merged with bright-field images. Scale bars, 50 μm. (TIFF) [file pone.0266982.s003.tiff]
